# G protein-coupled receptor 183 mediates the sensitization of Burkitt lymphoma tumors to CD47 immune checkpoint blockade by anti-CD20/PI3Kδi dual therapy

**DOI:** 10.3389/fimmu.2023.1130052

**Published:** 2023-04-21

**Authors:** Marcelo Lima Ribeiro, Núria Profitós-Pelejà, Juliana Carvalho Santos, Pedro Blecua, Diana Reyes-Garau, Marc Armengol, Miranda Fernández-Serrano, Hari P. Miskin, Francesc Bosch, Manel Esteller, Emmanuel Normant, Gael Roué

**Affiliations:** ^1^Lymphoma Translational Group, Josep Carreras Leukemia Research Institute, Badalona, Spain; ^2^Laboratory of Immunopharmacology and Molecular Biology, Sao Francisco University Medical School, Braganca Paulista, São Paulo, Brazil; ^3^Cancer Epigenetics Group, Josep Carreras Leukemia Research Institute, Badalona, Spain; ^4^Department of Biochemistry and Molecular Biology, Autonomous University of Barcelona, Barcelona, Spain; ^5^TG Therapeutics, New York, NY, United States; ^6^Department of Hematology, Vall d’Hebron University Hospital, Barcelona, Spain; ^7^Experimental Hematology, Vall d’Hebron Institute of Oncology, Barcelona, Spain; ^8^Centro de Investigación Biomédica en Red de Cáncer (CIBERONC), Instituto de Salud Carlos III, Barcelona, Spain; ^9^Instituciò Catalana de Recerca i Estudis Avançats (ICREA), Barcelona, Spain

**Keywords:** B-NHL, immune checkpoint blockade, drug combination, ADCP, M1 macrophage, 3D spheroid, CAM assay, inflammatory receptor

## Abstract

**Background:**

Immunotherapy-based regimens have considerably improved the survival rate of B-cell non-Hodgkin lymphoma (B-NHL) patients in the last decades; however, most disease subtypes remain almost incurable. TG-1801, a bispecific antibody that targets CD47 selectively on CD19+ B-cells, is under clinical evaluation in relapsed/refractory (R/R) B-NHL patients either as a single-agent or in combination with ublituximab, a new generation CD20 antibody.

**Methods:**

A set of eight B-NHL cell lines and primary samples were cultured *in vitro* in the presence of bone marrow-derived stromal cells, M2-polarized primary macrophages, and primary circulating PBMCs as a source of effector cells. Cell response to TG-1801 alone or combined with the U2 regimen associating ublituximab to the PI3Kδ inhibitor umbralisib, was analyzed by proliferation assay, western blot, transcriptomic analysis (qPCR array and RNA sequencing followed by gene set enrichment analysis) and/or quantification of antibody-dependent cell death (ADCC) and antibody-dependent cell phagocytosis (ADCP). CRISPR-Cas9 gene edition was used to selectively abrogate GPR183 gene expression in B-NHL cells. In vivo, drug efficacy was determined in immunodeficient (NSG mice) or immune-competent (chicken embryo chorioallantoic membrane (CAM)) B-NHL xenograft models.

**Results:**

Using a panel of B-NHL co-cultures, we show that TG-1801, by disrupting the CD47-SIRPα axis, potentiates anti-CD20-mediated ADCC and ADCP. This led to a remarkable and durable antitumor effect of the triplet therapy composed by TG-1801 and U2 regimen, *in vitro*, as well as in mice and CAM xenograft models of B-NHL. Transcriptomic analysis also uncovered the upregulation of the G protein-coupled and inflammatory receptor, GPR183, as a crucial event associated with the efficacy of the triplet combination. Genetic depletion and pharmacological inhibition of GPR183 impaired ADCP initiation, cytoskeleton remodeling and cell migration in 2D and 3D spheroid B-NHL co-cultures, and disrupted macrophage-mediated control of tumor growth in B-NHL CAM xenografts.

**Conclusions:**

Altogether, our results support a crucial role for GPR183 in the recognition and elimination of malignant B cells upon concomitant targeting of CD20, CD47 and PI3Kδ, and warrant further clinical evaluation of this triplet regimen in B-NHL.

## 1 Introduction

Immunotherapy regimens based on checkpoint inhibitors, tumour vaccination, immune cell-based therapy and cytokines, utilize the power and specificity of the host’s immune system against cancer and have become one of the most promising therapeutic interventions in oncology (1). This holds particularly true in B-cell lymphoma, with the considerable advances recently achieved by PD1/PD-L1, CAR-T cell therapies and CD3/CD20 bispecific antibodies, in heavily pre-treated patients (2). Prior to these new approaches, combining therapeutic (i.e. anti-CD20) antibodies with conventional chemotherapy have marked a milestone in the treatment of these diseases, although treatment toxicity and counteracting effects of the tumor-supportive microenvironment that affect the efficacy of immunotherapies, remained a challenge in a significant proportion of patients (3). Among the entities that benefited the most from the venue of anti-CD20 agents, Burkitt lymphoma (BL) is a rare and highly malignant type of B-cell lymphoma that accounts for approximately 50% of non-Hodgkin lymphoma (B-NHL) in children and adolescents (4). In 85% of the cases, BL is molecularly defined by the overexpression of the oncogene MYC caused by the translocation of 8q24 region to the immunoglobulin heavy chain locus (14q32) (5). Among the different immunological targets currently under evaluation on BL, cluster of differentiation 47 (CD47), also known as integrin-associated protein (IAP), is a cell surface receptor that is part of the immunoglobulin superfamily, and which interacts with the macrophage receptor signal regulatory protein-alpha (SIRPα). This interaction sends a “do-not-eat-me” signal to macrophages, which mediates immune evasion in several types of cancers, from both hematological and non-hematological origin (6). High levels of CD47 have indeed been observed in both lymphoid and myeloid neoplasms, in which this factor is both an adverse prognostic indicator and a valid anti-cancer target with several therapeutic antibodies currently being tested in clinical trials. In B-cell lymphoma, these trials frequently involve a combination with anti-CD20 therapy, to ensure a proper engagement of the Fc receptors at the surface of macrophages and natural killer (NK) effector cells. The anti-CD20 mAb rituximab has been the most common IgG1 antibody tested in this setting, and has demonstrated combinatorial activity in both indolent and aggressive entities (7, 8). However, as CD47 is widely expressed on the surface of a broad range of cell types, including erythrocytes and platelets, a major limitation of CD47 blocking agents is the target-mediated drug disposition and the potential side effects, which include anaemia or thrombocytopenia.

TG-1801 (also known as NI-1701) is a fully human IgG1 anti-CD47xCD19 bispecific antibody with the whole spectrum of Fc-mediated effector function, and that binds CD47 with sub-micromolar affinity and CD19 with a sub-nanomolar affinity. This thousand-fold difference between its affinity to these antigens allows TG-1801 to bind selectively to CD19-positive B cells *in vitro* and *in vivo*, including malignant B cells, but not CD19-negative red blood cells or platelets (9–11). TG-1801 is currently being tested clinically as a single agent and in combination with the glyco-engineered CD20 antibody, ublituximab, in patients with R/R B-cell lymphoma (NCT04806035).

In parallel, pre-clinical and clinical data have provided a rationale for the combination of anti-CD20 antibody with PI3Kδ antagonists in B-cell malignancies, mediated by the capacity of these latter to potentiate CD20-mediated direct cell death (12). Clinical efficacy and safety of this combinatorial approach has been confirmed in R/RB-NHL and chronic lymphocytic leukaemia (CLL) patients (13). Although the value of associating CD20 mAb therapy to CD47 modulating agents has been well established in aggressive B-cell lymphoma, both preclinically and clinically, two questions remained unanswered. First, we still don’t known whether the addition of PI3K-targeting agents to those immunotherapeutic regimens that contain checkpoint blockers could impact the efficacy of these latter. Second, beside rituximab which mainly depletes B cell by complement fixation, it would be interesting to assess in these settings the efficacy of new generation anti-CD20 antibodies harbouring a glycoengineering Fc region and a higher capacity to elicit ADCC. To investigate these two issues, we studied the effect of the B-cell specific, CD47/CD19 immune checkpoint blocker, TG-1801, in association with anti-CD20/PI3Kδi dual therapy in different *in vitro* and *in vivo* models of BL, as a disease model of aggressive B-NHL.

## 2 Materials and methods

### 2.1 Cell lines

Three BL (Raji, Daudi, Ramos), two diffuse large B cell lymphoma (DLBCL) (Pfeiffer, and Karpas-422 (Karpas)), one follicular lymphoma (FL) (RL), and one T-cell acute lymphoblastic leukemia (Jurkat) cell lines were used in this study. Cells were grown in Advanced-RMPI 1640 medium supplemented with 5% heat-inactivated FBS, 2 mmol/L glutamine, and 50 µg/mL penicillin-streptomycin (Thermo Fisher). All cultures were routinely tested for mycoplasma infection by PCR and the identity of all cell lines was verified by using AmpFISTR identifier kit (Thermo Fisher).

### 2.2 Occupancy assay

Cytofluorimetric quantification of CD47 and CD19 membrane levels was carried out in a panel of 10 B-NHL cell lines. Cells were stained with phycoerythrin (PE)-labelled anti-CD47 or anti-CD19 antibodies (Becton Dickinson) and the absolute number of membrane-bound molecules of CD47 or CD19 was estimated using QuantiBRITE PE beads (BD Biosciences) on a FACSCanto II (Becton Dickinson). Data were analysed using FlowJo software package (TreeStar, USA).

For the detection of unbound surface CD47, Raji (CD19+), or Jurkat (CD19-) cells were stained with a PE-labelled anti-CD47 (B6H12 clone) or isotype control antibody (BD Biosciences). Cells were pre-treated for 1 h with TG-1801 or an anti-human CD47 (B6H12 clone) control antibody. For quantification, a total of 10.000 events were acquired on a FACSCanto II (Becton Dickinson). Relative median fluorescence intensity (RMFI) was calculated using FlowJo software package as the ratio between CD47 and control signal intensity. Shown are the percentages of occupancy, defined as the decreases in CD47 RMFI ratios evoked by anti-CD47-treatment, using untreated cells as a calibrator. B6H12 clone was used as a CD19-independent positive control of CD47 occupancy.

### 2.3 Peripheral blood mononuclear cells isolation and macrophage polarization

Peripheral blood mononuclear cells (PBMCs) were purified by standard Ficoll-Hypaque (GE Healthcare) gradient centrifugation from buffy coats of human healthy donors and cultured freshly in Advanced-RMPI 1640 medium supplemented with 5% heat-inactivated FBS, 2 mmol/L glutamine, 50 µg/mL penicillin-streptomycin (Thermo Fisher).

RosetteSep™ Human Monocyte Enrichment Cocktail (Stemcell Technologies) was used to purify human monocytes from buffy coats following manufacturer specifications. For M1 or M2 macrophage polarization, the selected monocytes were cultured in complete Advanced-RMPI 1640 supplemented with either 20 ng/mL human GM-CSF (PeproTech), for M1 differentiation, or 20 ng/mL human M-CSF (PeproTech), for M2 differentiation, and incubated for 6 days. On day 6 M0 macrophages were activated with 100 ng/mL human IFN-γ (PeproTech) and 50 ng/mL LPS, for M1 macrophage polarization for 24 h.

### 2.4 Antibody-dependent cell-mediated cytotoxicity and phagocytosis assays

ADCC activity was assessed in B-cell lymphoma cell lines co-cultured for 4 hours with freshly obtained PBMCs (1:10, target:effector), in the presence of 10 ng/mL TG-1801 +/- U2 dual assets (10 µg/mL ublituximab + 1 µM umbralisib), using and a lactate deshydrogenease (LDH) release assay (Roche). Relative ADCC was calculated using the following formula: ADCC percentage = [(sample release – spontaneous release)/(maximal release – spontaneous release)]*100.

Spontaneous release, corresponding to target cells incubated with effector cells without antibody, was defined as 0% cytotoxicity, with maximal release (target cells lysed with 1% Triton X-100) defined as 100% cytotoxicity. The average percentage of ADCC and standard deviations of the triplicates of each experiment were calculated.

ADCP activity was assessed in B-cell lymphoma cell lines co-cultured for 4 hours with M1-polarized macrophages (1:5, target:effector), in the presence of 10 ng/mL TG-1801 +/- U2 dual assets (10 µg/mL ublituximab + 1 µM umbralisib), using and the pHrodo-stained B cells (IncuCyte^®^ pHrodo^®^ Red Cell Labelling Kit for Phagocytosis, Sartorius). Following phagocytosis assay, the non-phagocytosed cells were removed by washing with PBS 2–3 times and phagocytosis was analysed by fluorescent microscopy on an EVOS M5000 Cell Imaging Systems (Thermo Fisher).

### 2.5 Xenograft mouse model and tumor immunophenotyping

Eight-week-old NOD/SCID IL2Rγ-null (NSG) male and female mice (Janvier Labs) were subcutaneously injected with Raji cells and after two weeks tumor-bearing mice were randomized using GraphPad Prism 9.0 software (GraphPad Software, Inc) and assigned to one of the following treatment arms (8-6 mice per group): TG-1801 (5 mg/kg, qw), ublituximab (5 mg/kg, qw) + umbralisib (U2) (150 mg/kg, bid), or the triplet (TG-1801 + U2), or an equal volume of vehicle for 17 days. Tumour volumes were measured each 2-3 days with external callipers. The number of animals used in each of the experimental groups is based on the literature and previous results from the group (14). Immunohistochemical staining of representative tumor specimens (n=3 per group) was performed using anti-CD20 (Sigma), anti-GPR183 (Santa Cruz), anti-F4/80 (Abcam), anti-Histone H3-pSer10 (Abcam) and anti-CD56/NCAM-1 (Abcam) primary antibodies, as previously described (14). Preparations were evaluated using an Olympus BX53 microscope and MicroManager software (Fiji, Plugin).

Immunohistochemical signal intensity was quantified in at least 5 pictures of two representative tumor specimens from the Raji xenograft model, using QuPath v.0.2.3 software developed at Queen’s University (Belfast, Northern Ireland) (15). Cell detection was conducted using QuPath’s built-in “Positive cell detection” by calculating the percent of positively stained cells in each field.

### 2.6 Chicken embryo chorioallantoic membrane model

Fertilized white Leghorn chicken eggs were purchased from Granja Santa Isabel, S. L. (Córdoba, Spain) and incubated for 9 days at 37°C with 55% humidity. At day 9 of their embryonic development, eggs were cleaned with ethanol 70° and a window of an approximate 2 cm-diameter was drilled on top of the air chamber of the eggshell. Then, one million Raji-GPR183^WT^ (n=10) or Raji-GPR183^KO^ (n=10) Raji cells per egg were resuspended in 25 µL RPMI medium containing 10% FBS and 100 U/mL penicillin and streptomycin (Thermo Fisher) and 25 µL Matrigel (BD Biosciences). The mix was incubated for 15 min at 37°C and subsequently implanted into the CAM. The window was then covered with a sterile tape and the eggs were placed back in the incubator. At days 12 and 14 of their embryonic development, 10 ng/mL TG-1801 +/- U2 dual assets (10 µg/mL ublituximab + 1 µM umbralisib) or vehicle diluted in RPMI medium were administered topically on the tumor-bearing CAMs. On the 15th day of development (6 days post-implantation), chick embryos were sacrificed by decapitation. Tumors were excised and carefully weighed to determine their mass. Immunohistochemical detection and quantification of CD20 and GPR183 were carried out as above, in representative tumor specimens (n=3 per group).

### 2.7 RNA sequencing analysis

Two BL cell lines (Daudi and Raji) and two BL primary samples were co-cultured with the bone marrow stromal (BMSC) cell line, StromaNKterts, M2-polarized macrophages and PBMCs (4:1:1:1) in the presence of 10 ng/mL TG-1801 +/- U2. After a 24h incubation, CD20+ target cells were isolated using the EasySep Human Biotin Positive Selection Kit II (StemCell Technologies) and the biotinylated anti-CD20 antibody (BioLegend). Purified CD20+ cells, together with representative bulk Raji xenografts with > 95% tumor B cells were subjected to RNA-seq analysis according to previous procedures (14). Volcano plot showing the most relevant significantly differentially expressed genes between triplet and TG-1801 treatments, with |Log2 fold change| > 1.5 and p-adj value < 0.01 (red dots). Grey, green and blue dots identified genes with insignificant transcriptional and/or statistical variation. Briefly, the raw fastq RNAseq files of each condition were quality checked and gene expression was estimated using Salmon software (https://combine-lab.github.io/salmon/). Differential expression analysis was then carried out using the negative binomial distribution (DESeq2 software, https://bioconductor.org/packages/release/bioc/html/DESeq2.html), accounting for and filtering the effects of the respective controls. Sequencing data have been deposited at the Gene Expression Omnibus (GEO) of the National Center for Biotechnology Information and are accessible through GEO Series accession GSE199413 at (https://www.ncbi.nlm.nih.gov/geo/query/acc.cgi?acc=GSE199413).

Purified CD20+ cells, together with representative Raji xenografts were subjected to RNA extraction and qPCR validation. Briefly, total RNA was extracted using TRIZOL (Thermo Fisher) following manufacturer’s instructions. One microgram of RNA was retrotranscribed to complementary DNA using Moloney murine leukemia virus reverse transcriptase (Thermo Fisher) and random hexamer primers (Roche). mRNA expression was analyzed in triplicate by quantitative real-time PCR and the relative expression of each gene was quantified by the comparative cycle threshold method (ΔΔC_t_) *β-actin* (Fw: GACGACATGGAGAAAATCTG, Rv: ATGATCTGGGTCATCTTCTC) were used as an endogenous control. The sequences used for the primers are the following *GPR183* (Fw: GACTGGAGAATCGGAGATGC, Rv: CAGCAATGAAGCGGTCAATA), *CCL20* (Fw: CCAATGAAGGCTGTGACATCA, Rv: AGTCTGTTTTGGATTTGCGCA), *IL8* (Fw: AAGGAAAACTGGGTGCAGAG, Rv: GCTTGAAGTTTCACTGGCATC), *CD68* (Fw: CCTCCAGCAGAAGGTTGTCT, Rv: CGAAGGGATGCATTCTGAGC), *CCL4* (Fw: TTCCTCGCAATTTGTGGTA, Rv: GCTTGCTTCTTTTGGTTTGG), *CCL7* (Fw: TGG AGA GCTACAGAAGGACCA, Rv: GGGTCAGCACAGATCTCCTT), *CXCL1* (Fw: CATCCAAAGTGTGAACGTGAA, Rv: CTATGGGGGATGCAGGATT), *CXCL3*, (Fw: CAAAGTGTGAATGTAAGGTCCCC, Rv: CGGGGTTGAGACAAGCTTTC) and *CXCL10* (Fw: CCTGCAAGCCAATTTTGTCCA, Rv: TGGCCTTCGATTCTGGATTCA).

### 2.8 Generation of Raji-GPR183^KO^ cells

The generation of a CRISPR-Cas9 gene-editing tool was employed to edit the Raji parental cells line to create GPR183 knockout. 0.5 x 10^6^ cells were electroporated on a Nucleofector II device (program A032, Lonza) with 36 pmol SpCas9 Nuclease V3, 44 pmol CRISPR-Cas9 tracRNA ATTO 550, 44 pmol Alt-R CRISPR-Cas9 crRNA Hs.Cas9.GPR183.1.AA (GPR183^KO^ 5´- CAATGAAGCGGTCAATACTC AGG -3`) (IDT-Integrated DNA Technologies). GPR183^KO^ cells were resuspended in 96-well plates with a limiting dilution of 0.3 cells per well. Two GPR183^KO^ biallelic clones were confirmed by Sanger Sequencing and western blot. Raji-GPR183^KO^ clones are available upon request.

### 2.9 Western blot analysis

Total protein extracts were obtained from cell lines and tumor specimens using RIPA (Sigma-Aldrich) buffer and subjected to SDS-PAGE. Membrane-transferred proteins were revealed by incubating with primary and secondary antibodies followed by chemiluminescence detection using the ECL system (Pierce) and a Fusion FX imaging system (Vilber Lourmat). Band intensity was quantified using Image J software and normalized to housekeeping protein (GAPDH). Values were referred to the indicated control and added below the corresponding band. If not otherwise specified, representative data from n=2 experiments are shown.

### 2.10 3D multicellular spheroid generation

One hundred thousand Raji-GPR183^WT^ or Raji-GPR183^KO^ (clone#1) cells were then stained with Hoechst 33342 blue dye (Invitrogen) and cultivated in a conditional medium with 25.000 StromaNKtert-GFP cells for 2 days to generate the BL 3D spheroids. Then, 25,000 M1-macrophages were stained with PKH26 red‐fluorescent dye and added to 3D spheroid in the presence or absence of 10 ng/mL TG-1801 +/- U2 (10 µg/mL ublituximab + 1 µM umbralisib) for one more day. The M1-macrophages infiltration was evaluated by live-cell red fluorescence on the EVOS M5000 Cell Imaging Systems.

### 2.11 Transwell migration assay and F-actin staining

Briefly, Raji-GPR183^WT^, Raji-GPR183^KO^ (clone#1) and Raji parental cells exposed to the GPR183 inhibitor NIBR189 (Sigma-Aldrich) were cultured for 1 h in culture medium w/o FBS but supplemented with 0.5% bovine serum albumin (Sigma-Aldrich), in the presence or absence of 10 ng/mL TG-1801 +/- U2 combination, and analyzed for CXCL12-dependent chemotaxis, as previously described (16). Values were referred to cells cultured without CXCL12. F-actin levels were assessed after exposure to TG-1801 +/- U2, followed by staining with a TRITC-labelled phalloidin and direct red fluorescence recording.

### 2.12 Ethics

Animals were handled following protocols approved by the Animal Ethics Committee of the University of Barcelona (registry num. 38/18). Institutional Review Board approvals for the study protocol (ref PI-20-040), amendments, and written informed consent documents from BL patients and healthy donors were obtained prior to study initiation. Study procedures were conducted in accordance with the Declaration of Helsinki. Buffy coats were provided by the Blood and Tissue Bank of Catalonia (agreement NE-A1-IJC).

### 2.13 Statistical analysis

Presented data are the mean ± SD or SEM of 3 independent experiments. All statistical analyses were done by using GraphPad Prism 9.0 software (GraphPad Software). Comparison between two groups of samples was evaluated by nonparametric Mann–Whitney test to determine how the response is affected by 2 factors. Pearson test was used to assess the statistical significance of correlation. Results were considered statistically significant when *p*-value < 0.05.

## 3 Results

### 3.1 Anti-CD20/PI3Kδi dual asset cooperates with CD47 blockade therapy in CD19+ B-cell tumors

**Figure 1:**
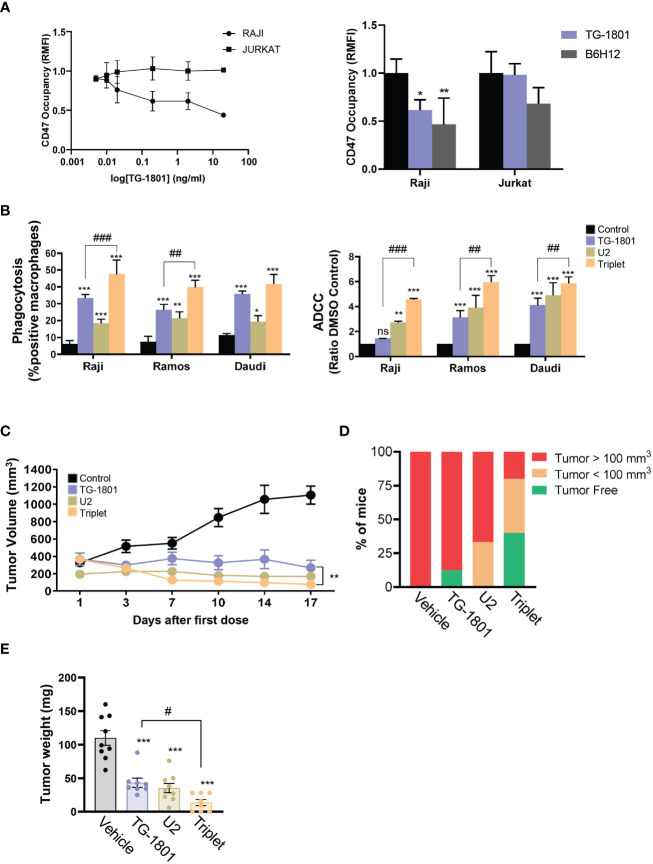
U2 regimen cooperates with CD47 blockade in *in vitro* and *in vivo* models of B-cell lymphoma. **(A)** FACS-mediated CD47 occupancy assay in the CD19+ Burkitt lymphoma cell line Raji, and in the CD19- T-ALL cells Jurkat, treated for 1h with different doses of TG-1801 or B6H12. Results are expressed in Relative median fluorescence intensity (RMFI) of the PE-labelled anti-CD47 antibody. Left panel is a dose-occupancy curve of Raji and Jurkat cell lines with different doses of TG-1801. Right panel is a comparison between TG-1801 and B6H12 at 2µg/ml (n=3). **(B)** ADCP (left panel) and ADCC (right panel) activities were assessed in three representative BL cell lines (N=3). Values are expressed as mean ± SD. **(C)** NOD/SCID IL2Rγ-null (NSG) mice were inoculated subcutaneously with 10^7^ Raji cells and after 14 days, tumour-bearing animals (n=6-8 mice per group) were orally dosed for 17 days with TG-1801 (5 mg/kg, qw), ublituximab (5 mg/kg, qw) + umbralisib (U2) (150 mg/kg, bid), the triplet (TG-1801 + U2), or an equal volume of vehicle. Tumour volumes were measured each 2-3 days with external callipers. **(D)** Mice with no tumour or low tumour size were kept alive for another 35 days. At day 52 all the mice either tumour-free (green) or bearing a small tumour (orange, < 100 mm^3^) were alive. The triplet combo group showed a higher number of tumour-free or low tumour burden-bearing mice. **(E)** Weight recording of Raji CAM-derived tumour at day 15 exposed to TG-1801, U2 or triplet combination (n=8-10 replicates per treatment group). * *p*<0.05, ** *p*<0.01, *** *p*<0.001, when compared to control group. ^#^ p<0.05, ^##^ *p*<0.01 and ^###^ *p*<0.001 when compared to TG-1801 alone. ns, non-significant.

The CD47/CD19 bispecific antibody TG-1801 has recently been shown to trigger *in vitro* response against CD19+ tumors; however these studies did not determine the lowest effective concentrations of this antibody, which may be further used in combination approaches (11). To determine the best working concentrations of TG-1801 *in vitro*, we developed a CD47 occupancy assay using the BL cell line Raji. In this assay, the bispecific CD47/CD19 antibody reached a similar CD47 occupancy compared with the first-in-class CD47 blocking mAb, B6H12 (17) (**Figure 1A**, right panel and **Supplemental Figure S1A**). This difference may potentially be explained by the lower level of expression of CD19 compared to CD47 in the BL cell line (data not shown). As expected, TG-1801, but not B6H12-mediated target occupancy, was highly dependent on CD19 expression, as shown when using the T-ALL-derived, CD19-negative, Jurkat cells, in which no significant CD47 blockade was detected with TG-1801 at doses as high as 2 µg/mL, contrasting with the sustained binding of B6H12 at the same concentration (**Figure 1A**, right and left panels). To evaluate the rationale for combining TG-1801 with ublituximab, a type 1 chimeric anti-CD20 IgG1κ mAb and the PI3Kδ inhibitor umbralisib (U2 dual asset), Raji cells were exposed to these agents either alone or in combination, and cultured in the presence of M1-polarized macrophages or primary circulating PBMCs from healthy donors as a source of effector cells, to assess ADCP or ADCC induction, respectively. As shown in **Supplemental Figure S1B**, the U2 combination triggered the highest levels of ADCC and ADCP, displaying a 3-fold increase over the control and a significant increase in antibody-dependent cell death compared to both umbralisib and ublituximab alone. Subsequently, U2 combo was directly tested in association with TG-1801 at the previously determined dose, and ADCP and ADCC levels were evaluated as above in a panel of n=6 human B-cell lymphoma cell lines from either BL (Raji, Daudi and Ramos; **Figure 1B**), DLBCL (Pfeiffer and Karpas-422; **Supplemental Figure S1C**), or FL (RL; **Supplemental Figure S1C**) origin. Although both biological processes were visibly potentiated in almost all of the cell lines exposed to the triplet treatment, a comparative analysis demonstrated that, in BL cell lines both TG-1801 pro-cytotoxic and pro-phagocytic activities were potentiated (1.4- to 3.2-fold) by U2 addition, whereas only a barely additive effect of the triplet combination on ADCC was observed in DLBCL- and FL-derived cell lines. Of note, this effect appeared to be related to neither the expression levels of CD47, nor to CD47/CD19 ratios (**Supplemental Figure 1D**), in accordance with previous reports (10). To carry out an *ad-hoc* drug-drug interaction analysis, a new ADCP analysis was performed in the 3 BL cell lines exposed to increasing doses of TG-1801 (5-10-20 ng/ml), ublituximab (1-2-4 ng/ml) and umbralisib (0.5-1-2 µM), and the drug combination indexes (CI) were calculated using the Chou and Talalay’s algorithm. As shown on **Supplemental figure S1E**, for 20 out of the 27 different TG-1801/U2 combinations evaluated, the CI value was ≤ 0.8, indicative of synergistic drug interaction.

Based on these results, we evaluated the activity of the TG-1801+U2 triplet combo in a BL (Raji-derived) mouse xenograft. In line with our abovementioned observation, in tumor-bearing mice TG-1801 and U2 exerted notable anti-lymphoma activities, with 76% and 89% tumor growth inhibition (TGI) at day 17 of treatment, respectively (**Figure 1C**). Nonetheless, the activity of the triplet was maximum as early as 7 days of treatment, reaching a 93% TGI at a time point where TG-1801 monotherapy was almost ineffective. This superior efficacy was maintained during the whole treatment schedule (**Figure 1C**), with no detectable toxicity (data not shown). Of special interest, 2 out of 5 mice (40%) remained tumor-free 35 days after the last dose in the triplet arm, compared to the reduced rate of prolonged remission (12.5%) in the TG-1801 group (**Figure 1D**).

To pinpoint the effect of the triple combination, an immunocompetent chicken chorioallantoic membrane (CAM)-derived xenograft Raji model (18) was carried out (see experimental workflow in **Supplemental Figure S1F**). Similarly, to the mouse model, in the Raji-derived CAM assay, both TG-1801 and U2 treatments inhibited 70% of tumor growth and the activity of the triplet was significantly higher (86% TGI, **Figure 1E**).

Altogether, these *in vitro* and *in vivo* data suggested that the combination of CD47 checkpoint blockade therapy to dual CD20/PI3K targeting evokes a mechanism that promotes a stronger and prolonged innate immune response when compared to each agent used separately.

### 3.2 GPR183 is upregulated in response to CD47/CD20/PI3Kδi triplet treatment

**Figure 2:**
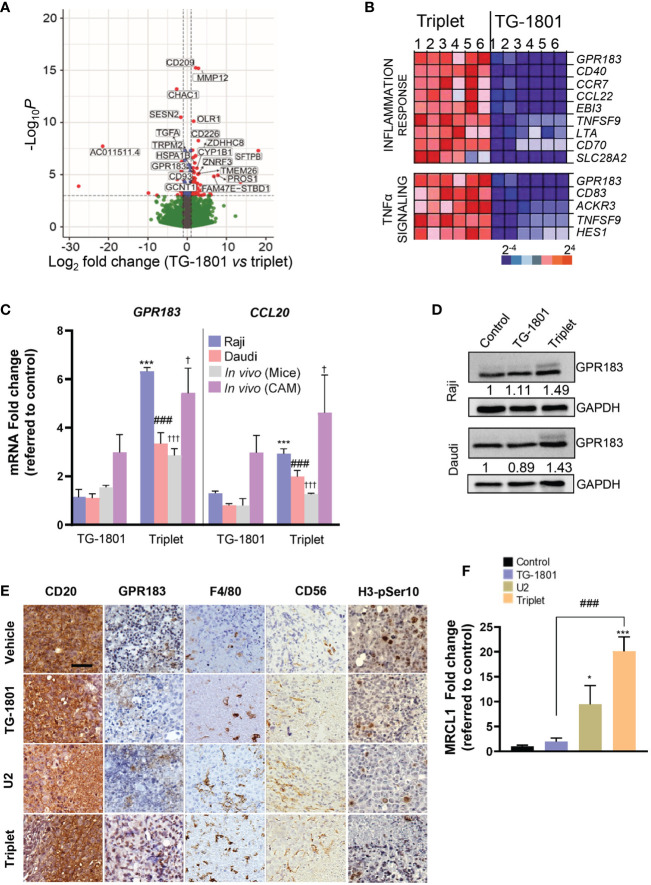
Upregulation of GPR183 is a hallmark of U2 and TG-1801 combinational effect *in vitro* and *in vivo*. **(A)** Volcano plot showing the most relevant significantly differentially expressed genes between triplet and TG-1801 treatments in n=6 BL samples (two cell lines, two primary samples and two representative Raji xenografts specimens). Genes undergoing a similar modulation in all the *in vitro* and *in vivo* models (n=20) have been labelled. **(B)** GSEA-mediated identification of the main enriched gene sets in the triplet-treated samples compared to the samples treated with TG-1801. Samples were sorted from left to right: 1-Raji, 2-Daudi, 3-4- BL primary samples and 5-6- CD20+ cells sorted from 2 representative Raji xenograft specimens. **(C)** qPCR determination of *GPR183* and *CCL20* transcript levels in Raji and Daudi cell lines exposed to TG-1801 or to the triplet therapy as previously, and in the two Raji *in vivo* (mouse and CAM) models dosed with the different agents. Values are referred to untreated Raji cultures. **(D)** Immunoblot detection and densitometric quantification of GPR183 protein levels (SantaCruz, #sc-514342) in BL cell lines exposed to TG-1801 +/- U2 dual asset, using GAPDH as a loading control. Values are normalized to control untreated cells. For each cell line, shown is a representative experiments out of three. **(E)** Immunohistochemistry (IHC) labelling of CD20 (Clone L26, Sigma-Aldrich), GPR183 (Clone G-12, Santa Cruz), F4/80 (Clone SP115, Abcam), Histone H3-pSer10 (Clone E173, Abcam) and CD56/NCAM-1 (Clone EPR1827, Abcam) in tissue sections from tumour specimens (shown are pictures from 1 out of 3 representative specimens) Scale bar: 50 µm. **(F)** *MRCL1* transcript levels in the Raji-derived CAM model. Data are presented in fold-change related to the control (N=8-10) according to the different treatment regimens. * *p*<0.05, *** *p*<0.001, ^###^ *p*<0.001, and ^†^ *p*<0.05 and ^†††^ *p*<0.001 when compared to control group in Raji (*in vitro*), Daudi (*in vitro*) and Raji (*in vivo*) models, respectively. ns, non-significant.

To uncover the mechanisms underlying the superior effect of the triplet *vs* anti-CD47/CD19 monotherapy, transcriptomic analyses were carried out on a set of six samples that included Raji mouse xenograft tumors (n=2) and CD20+ cells isolated from Raji, Daudi and two adult sporadic BL primary samples (one case with abdominal involvement and another case with bone marrow involvement), co-cultured with the bone marrow-derived stromal cell line, stromaNKtert (19), M2-polarized primary macrophages, primary circulating PBMCs, in the presence of either TG-1801 or TG-1801+U2 triplet. As shown in **Figure 2A**, a total of 20 genes were significantly up- or down-regulated in the triplet compared to TG-1801 monotherapy, in all six samples and in both *in vitro* and *in vivo* settings. A gene set enrichment analysis (GSEA) of the same data identified inflammatory (NES=2.43, FDR=0) and TNFα-driven signatures (NES=2.43, FDR=0) as predominantly expressed upon treatment with the triplet combination, when compared to TG-1801 single agent therapy, suggesting that the stronger activity of the triplet was based on the activation of an immune-related antitumor effect. In the heatmap showing a set of genes strongly activated in all six samples in the triplet group (**Figure 2B**), the highest up-regulated gene in both signatures was the G protein-coupled receptor 183 (GPR183, also known as Epstein-Barr virus (EBV)-induced G protein-coupled receptor 2, EBI2). An increase in *GPR183* mRNA was confirmed by qPCR after a 4-hour and a 24-hour incubation of the four cell lines with TG-1801/U2 combo (**Figure 2C**; **Supplemental Figure S1G**). A comparable increase in GPR183 protein levels was also shown using western blotting in the Raji and Daudi cell lines subjected to the same treatments (+49% and +43%, respectively; **Figure 2D**), and by immunohistochemistry (IHC) in representative tumors from the two Raji xenograft models (mouse and CAM) dosed with the triplet regimen (**Figure 2E**; **Supplemental Figure S1H**).

GPR183 was first identified by sequence similarity as a GPCR induced by Epstein-Barr virus (EBV) infection. The upregulation of this pro-inflammatory receptor is associated with a better prognosis of DLBCL patients treated with the standard immunochemotherapeutic (R-CHOP) regimen, according to published gene array database (gse10846; R2: Genomics Analysis and Visualization Platform (http://r2.amc.nl; http://r2platform.com)). In addition, GPR183 plays an important role in B-cell motility and positioning during the germinal center reaction (20, 21). The gradient of its natural ligand, oxysterol, acts as a chemoattractant of GPR183+ cells. Interestingly, among a panel of eight genes identified besides *GPR183* in the two inflammation gene signatures, the transcript of another chemoattractant gene, *CCL20*, was upregulated by the triplet combination in all *in vitro* and *in vivo* models (**Figure 2C**). In addition, increased tumor infiltration of mouse and chicken macrophages in BL tumors, as revealed by IHC detection of the mouse antigen F4/80 and qPCR quantification of the chicken antigen *MRCL1* (22), was accompanied by a loss of histone H3-pSer10 nuclear staining, suggestive of a consistent reduction in tumor mitotic index in animals subjected to triplet therapy, when compared to TG-1801 and U2 treatment arms (**Figures 2E, F**).

### 3.3 GPR183 is required for B-cell trafficking and macrophage-dependent phagocytosis after the triplet treatment

**Figure 3:**
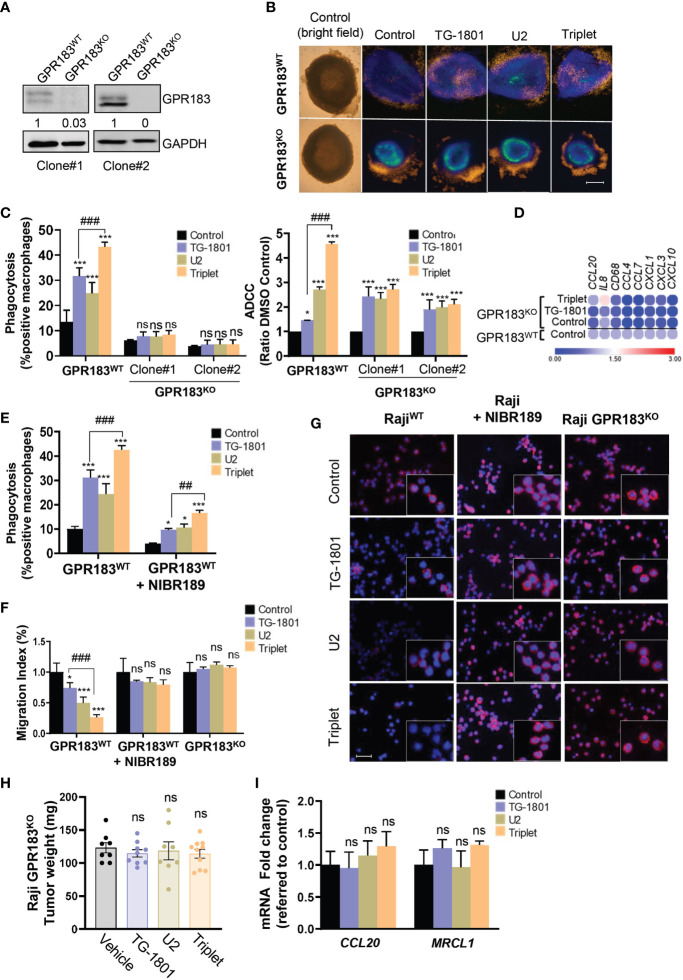
GPR183 is required for impaired B cell trafficking and tumour cell phagocytosis upon TG-1801/U2 triple combination therapy. **(A)** Immunoblot detection and densitometric quantification of GPR183 in both Raji-GPR183^WT^ and Raji-GPR183^KO^ cells, using GAPDH expression as a loading control. Values are referred to control, wild type cells. **(B)** Raji-GPR183^WT^ or Raji-GPR183^KO^ 3D spheroid in presence or absence of 10 ng/mL TG-1801 +/- U2 (10 µg/mL ublituximab + 1 µM umbralisib) for one more day. The infiltration of M1 macrophages was evaluated by live-cell red fluorescence. Scale bar: 500 µm. **(C)** Triggering of phagocytosis (left panel) and cell-mediated cytotoxicity (right panel) was assessed in Raji-GPR183^WT^ and Raji-GPR183^KO^ cultures upon quantification of engulfed B cell red fluorescence with pHrodo and LDH release assay, respectively. For each assay, shown are average values obtained from three independent replicates. **(D)** Raji-GPR183^WT^ and Raji-GPR183^KO^ were co-cultured with BMSCs, M2-polarized primary macrophages and PBMCs (4:1:1:1) and treated with vehicle, TG-1801 or the triplet combination for 24h. Then, purified CD20+ cells were subjected to RNA extraction and qPCR. Data are presented in fold-change related to the Raji-GPR183^WT^ control. **(E)** ADCP activities were assessed in Raji cells with or without the GPR183 inhibitor NIBR189 prior to treatment with 10 ng/mL TG-1801 +/- U2. Shown are average values from n=3 replicates. **(F)** The cell migration index of Raji-GPR183^WT^, Raji-GPR183^KO^ (clone #1) cells exposed to the GPR183 inhibitor NIBR189, in presence or absence of 10 ng/mL TG-1801 +/- U2 combination. Shown are average values from n=3 replicates. **(G)** F-actin levels were assessed in the different cultures exposed to TG-1801 +/- U2 as in **(E)**, followed by staining with a TRITC-labelled phalloidin and direct red fluorescence recording. Nuclei were counterstained with DAPI (blue) (shown are representative pictures from 1 out of 3 fields). Scale bar: 50 µm. **(H)** Tumour weights at day 15 for Raji GPR183^KO^ (N=8-10) exposed to TG-1801, U2 or triplet combination. **(I)** *CCL20* and *MRCL1* transcript levels in GPR183^KO^ tumours. Data are presented in fold-change related to the control (shown are median values from 8-10 replicates) according to the different treatment regimens. Values are expressed as mean ± SD. ^*^ *p*<0.05, ^***^ *p*<0.001, when compared to control group. ^##^ p<0.01 and ^###^ *p*<0.001 when compared to TG-1801 alone. ns, non-significant.

To investigate how the upregulation of GPR183 in cancer cells could impact their recognition and phagocytosis by M1 macrophages, two Raji-GPR183^KO^ single-clones (clone #1 and clone #2) were generated by CRISPR/Cas9 gene editing (**Figure 3A**) using previously described procedures (14). Cells from clone #1 were co-cultured for 24h with primary M1 macrophages and BMSCs in a conditioned medium to form functional 3D spheroids, as previously reported (23). Compared to their Raji-GPR183^wt^ counterparts, the Raji-GPR183^KO^ spheroids displayed a complete absence of M1 cell infiltration within the multicellular aggregates, both at basal levels and upon exposure to the triplet therapy (**Figure 3B**). Accordingly, ADCP activity was abrogated in the Raji-GPR183^KO^ cell cultures obtained from the two clones (**Figure 3C**, left panel) highlighting the critical role of GPR183 in the recruitment of macrophages after CD47/CD20/PI3Kδ triple targeting. ADCC was also compromised, although to a lower extent (**Figure 3C**, right panel). Supporting these results, global inflammatory signature, and especially *CCL20* gene overexpression, was not detected anymore in Raji-GPR183^KO^ (clone #1) co-cultures exposed to the triplet therapy (**Figure 3D**).

To evaluate whether a functional GPR183 was required for the triplet drug interaction, Raji cells were exposed for 1 hour to the GPR183 inhibitor NIBR189 (24), washed out, and co-cultured for 4 hours with M1-polarized macrophages, in the presence of U2 +/- TG-1801, and ADCP induction was quantified as described above. As shown in **Figure 3E**, the relative levels of phagocytosis were decreased by 3-fold after GPR183 pharmacological blockade in triplet-treated co-cultures, thus confirming the requirement of an active GPR183 receptor for the full activation of macrophage function.

Since GPR183 is a known antagonist of chemokine-mediated B cell migration (25), a transwell migration assay using recombinant CXCL12 as a chemoattractant was set up. The chemotaxis properties of CXCL12 were tested on the Raji parental cells, the Raji-GPR183^KO^ cells, and the NIBR189-pretreated cultures. **Figure 3F** shows that BL cell migration was significantly impaired by both U2 and TG-1801 treatments, and that the combination led to an accentuated (75%) inhibition of cell mobility. This effect was completely lost either after GPR183 pharmacological inhibition by NIBR189 or upon the genetic deletion of the receptor. Accordingly, F-actin polymerization was decreased by 70% in Raji cells exposed to the triplet treatment and this effect was counteracted by the absence of GPR183 (clone #1) or by its pharmacological blockade (**Figures 3G**, **S1I**), in agreement with previous studies highlighting the relevance of F-actin disruption in the anti-lymphoma effect of anti-CD47 antibodies (26). Finally, the crucial role of GPR183 in BL response to the triplet was confirmed *in vivo* in a Raji-GPR183^KO^ CAM model, in which TG-1801, U2, and the combination treatment failed to impact tumoral growth, with no modulation of *CCL20* expression and failed intratumoral infiltration of MRCL1+ macrophages (**Figures 3H, I**).

Altogether, these data strongly support that GPR183 upregulation is a prerequisite to the recruitment of macrophages and to the initiation of the inflammatory response, in Raji xenografts subjected to the triplet treatment.

## 4 Discussion

CD47 immune checkpoint inhibitors have emerged as promising immunotherapy in cancer treatment thanks to their ability to facilitate the concomitant inhibition of the “do-not-eat-me” signal and to boost antitumor T-cell response. Indeed, although CD47/SIRPα axis blockade was initially believed to lead to antitumor activity exclusively through ADCP (27), it was further demonstrated that targeting CD47 also promotes T cell–mediated elimination of immunogenic tumors (28, 29). Considering the relevance of the CD47/SIRPα axis in tumor progression, including under (chemo)-therapeutic pressure, several novel studies have demonstrated the potential of CD47-based combination therapies in different types of cancer (30). Among the most promising combinations, the synergistic FcR-dependent activation of phagocytosis by the anti-CD47 mAb Hu5F9-G4 associated to rituximab signaling has demonstrated great effectivity in B-NHL cell lines and primary samples (31), and has further shown promising clinical activity with few side effect in the phase Ib clinical trial NCT02953509 involving R/R DLBCL and FL patients (32). Similarly, bispecific antibodies that co-target CD47 and CD20 can induce selective phagocytosis of B-NHL cells, and prolong the survival time of mice transplanted with these tumors (33). This dual anti-CD20/anti-CD47 approach has provided convincing clinical results and is still being extensively evaluated, both pre-clinically and clinically, in B-cell lymphoma (7). Here, we present a new set of data that propose for the first time a biological rationale for the combination of the first-in-kind anti-CD47/CD19 bispecific antibody, TG-1801, with the clinically tested pair of the anti-CD20 antibody ublituximab with umbralisib, a PI3K inhibitor.

PI3Ks are a key component of cell machinery controlling cellular key pathways such as growth, proliferation, survival, migration and differentiation. Dysregulation of this pathway have been described in several types of cancer, including BL (34, 35). Expression of the PI3Kδ subunit is generally restricted to hematopoietic cells, and is essential for the development and expansion of normal and malignant B-cells (36). Preclinical data with different class-specific PI3K inhibitors have suggested that part of their antitumor activity relies on the modulation of the tumor microenvironment (TME) and to an enhancement of adaptive immunity, including an improved intratumoral macrophage infiltration, thereby increasing the efficacy of immune checkpoint blockade therapy (37–39). Our present data support for the first time the use of a PI3Kδ inhibitor to promote the remodeling of the tumor microenvironment *in vitro* and *in vivo*, for a better anti-CD47-mediated activation of macrophages. Indeed, we observed that the TG-1801/ublituximab/umbralisib triplet, when compared to TG-1801 monotherapy, significantly improved innate immunity *in vitro* and *in vivo*, with the potentiation of both macrophages and cytotoxic cell activity, although this latter at a lower extent. Unsurprisingly, most of this effect was due to TG-1801, which was already known to exert its antitumor effect by eliciting ADCP and ADCC (10) and to increase the phagocytosis of tumor cells by the macrophage and monocyte populations present in lymphoid TME (11). Of special interest, the superior antitumor activity of the triplet combination in mice resulted in a prolonged response with a higher number of tumor-free animals 35 days after the last dose. Transcriptomic and immunophenotypic analyses revealed that this sustained effect was closely linked to an enrichment in inflammatory gene signatures and an enhanced recruitment of macrophages and NK cells at the tumor site, being these two phenomena known prerequisites for full activity of immune checkpoint blockers (40).

By coupling phenotypic characterization to gene edition, we further propose that among the main pro-inflammatory genes activated in a set of *in vitro* and *in vivo* models of B-NHL that reconstitute most features of the lymphoid TME and the main aspects of B-NHL architecture, the GPR183 receptor represents a crucial regulator involved in the efficacy of the drug combination. GPR183 belongs to the rhodopsin family and is widely expressed in B, T, and dendritic cells (20, 40). This factor is responsible for regulating the positioning and migration of immune cells within secondary lymphoid organs (41, 42) and its deregulated activity or expression has been found in germinal center B-like DLBCL, FL, CLL and acute myeloid leukemia (43). Accordingly, we found that treatment-induced upregulation of GRP183 in BL tumors strongly affect F-actin polymerization in B cells, with the consequent impairment of CXCL12-dependent chemotaxis, a phenomenon that possibly render these latter more accessible to pro-inflammatory macrophages with the capacity to limit tumor growth (42). Finally, and in agreement with our finding although in another cellular context, loss-of-function mutation of *GPR183* or treatment with a GPR183 antagonist, have recently been associated with a reduced macrophage infiltration and a lower inflammatory cytokine production in the lungs of mice infected by respiratory viruses (44).

PI3K/Akt signaling was recently identified as the main downstream axis synergistically affected by co-exposure of malignant B cells to different anti-CD20 mAbs and to the PI3Kδ inhibitor idelalisib (45). However, in the case of BL and under determined experimental settings (i.e. upon exposure to immunomodulatory drugs), the anti-CD20 mAb rituximab was shown to promote EBV reactivation, thereby stimulating PI3K signaling (46). Although EBV intracellular signaling hasn’t been challenged here, one may hypothesize that, as a bona fide EBV-responsive gene, GPR183 may be upregulated by the simultaneous ligation of CD47 and CD20 by TG-1801 and ublituximab, rending at the same time these cells more susceptible to PI3Kδ blockade by umbralisib.

**Figure 4:**
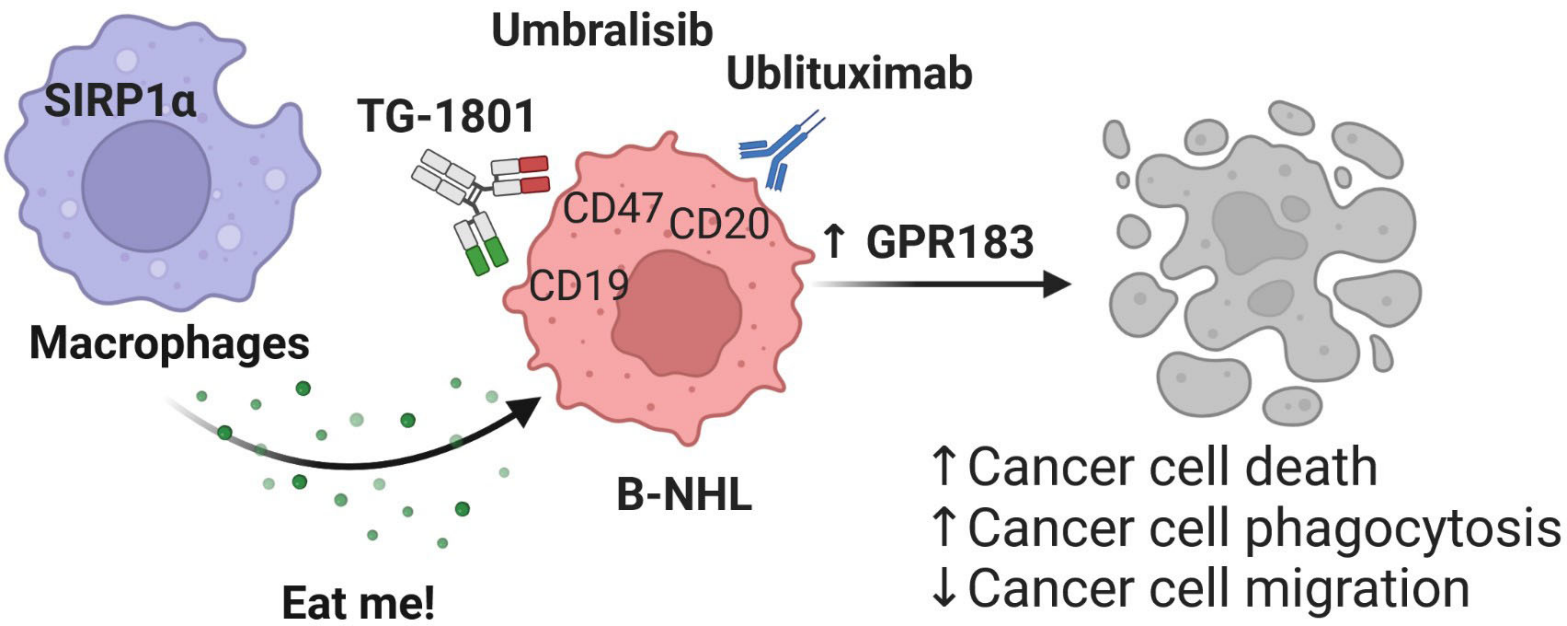
Mechanism of action of the TG-1801/U2 triplet combination therapy in BL. The novel CD47-CD19 bispecific antibody potentiates the anti-tumour activity of the ublituximab-umbralisib regimen through activation of the pro-inflammatory GPR183, thus promoting macrophage-dependent phagocytosis, B-cell cytoskeleton remodelling and B-cell motility.

In summary, our results support a role for GPR183 in the recognition and elimination *in vitro* and *in vivo* of malignant B cells by activated macrophages, upon concomitant targeting of CD20, CD47 and PI3Kδ in tumor cells (**Figure 4**). Future studies will be aimed at understanding whether GPR183 could constitute a *bona fide* biomarker for the activity of anti-CD47-based therapeutic regimens. Testing whether this discovery can be generalized to other agents from the same classes than those used here, is currently underway.

## Data availability statement

The datasets presented in this study can be found in online repositories. The names of the repository/repositories and accession number(s) can be found in the article/**Supplementary Material**.

## Author contributions

MR, NP-P and JS: Resources, investigation, methodology, writing–original draft. PB: Data curation, software, visualization. DR-G: Investigation, methodology. MA: Investigation. MF-S: Investigation. HM: Validation, writing review and editing. FB: Writing–review and editing. ME: Data curation, writing review and editing. EN: Conceptualization, resources, supervision, validation, writing–original draft, writing–review and editing. GR: Conceptualization, resources, supervision, funding acquisition, validation, investigation, visualization, writing–original draft, project administration, writing–review and editing. All authors contributed to the article and approved the submitted version.
