# Supplementary material for: G protein-coupled receptor 183 mediates the sensitization of Burkitt lymphoma tumors to CD47 immune checkpoint blockade by anti-CD20/PI3Kδi dual therapy
Source: Front Immunol. 2023 Apr 21;14:1130052. doi: 10.3389/fimmu.2023.1130052 (PMC10160608; doi:10.3389/fimmu.2023.1130052)
Supplement: Supplementary file 1 [file DataSheet_1.docx]

Supplementary Material

G Protein-Coupled Receptor 183 mediates the sensitization of Burkitt lymphoma tumors to CD47 immune checkpoint blockade by anti-CD20/PI3Kδi dual therapy

Marcelo Lima Ribeiro, Núria Profitós-Pelejà, Juliana Carvalho Santos, Pedro Blecua, Diana Reyes-Garau, Marc Armengol, Miranda Fernández-Serrano, Hari P. Miskin, Francesc Bosch, Manel Esteller, Emmanuel Normant, and Gael Roué*

*** Correspondence:** Gaël Roué: [groue@carrerasresearch.org](mailto:groue@carrerasresearch.org) .

# Supplementary Figure 1

**Supplementary Figure 1.**

A) FACS-mediated CD47 occupancy assay in the CD19+ Burkitt lymphoma cell line Raji, and in the CD19- T-ALL cells Jurkat, treated for 1h with different doses of TG-1801 (shown is a representative histogram from 1 out of 3 experiments). B) ADCP (left panel) and ADCC (right panel) activities were assessed in Raji cell line (n=3). Values are expressed as mean ± SD. C) ADCP (left panel) and ADCC (right panel) activities were assessed in RL, Pfeifer and Karpas cell lines (n=3). Values are expressed as mean ± SD. D) Cytofluorimetric quantification of CD47 and CD19 levels in a panel of 10 B-NHL cell lines. E) Combination index (CI) values were calculated in Raji, Daudi and Ramos cells exposed to different concentrations of each triplet component and subjected to ADCP assay. F) Scheme depicting the timeline of the chicken embryo chorioallantoic membrane (CAM) xenotransplant model. G) GPR183 upregulation was confirmed in these samples and compared to the 11 inflammatory genes extracted from GSEA analysis. Data are presented in fold-change related to the control (n=3). Clustering was performed using Morpheus (hierarchical, one minus Pearson correlation) available at https://software.broadinstitute.org/morpheus/. H) Immunohistochemistry (IHC) labelling of CD20 (clone L26, Sigma-Aldrich) and GPR183 (clone G-12, Santa Cruz), in tissue sections from GPR183wt tumor specimens (n=3). Scale bar: 50 µm. I) F-actin fluorescence intensity from Raji-GPR183WT, Raji-GPR183KO, and NIBR189-treated Raji cells in presence or absence of TG-1801 +/- U2.

**
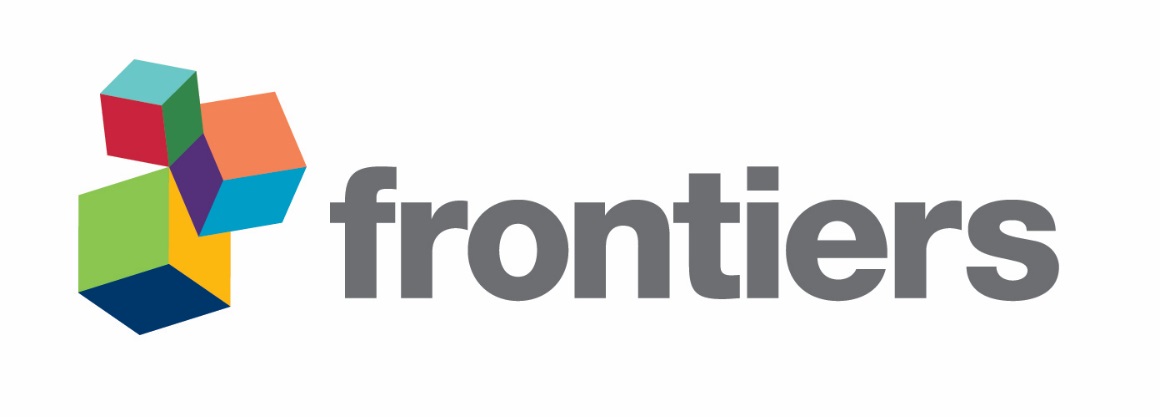
**
